# Trailblazers in cancer research: the next generation – the British Association of Cancer Research early-career conference

**DOI:** 10.1242/bio.060121

**Published:** 2023-10-17

**Authors:** Kyle K. Greenland, Kathryn A. F. Pennel, Giorgia Cioccoloni, Connor Rogerson, Francis M. Barnieh, Valerie Speirs

**Affiliations:** ^1^ Imperial Centre for Translational and Experimental Medicine, Department of Surgery and Cancer, Imperial College London, Hammersmith Hospital Campus, London W12 0NN, UK; ^2^ School of Cancer Sciences, Wolfson Wohl Cancer Research Centre, University of Glasgow, Glasgow G61 1BD, UK; ^3^ School of Food Science & Nutrition, Faculty of Environment, University of Leeds, Leeds LS2 9JT, UK; ^4^ Department of Biochemistry, University of Cambridge, Cambridge CB2 1GA, UK; ^5^ Institute of Cancer Therapeutics, Faculty of Life Sciences, University of Bradford, Bradford BD7 1DP, UK; ^6^ Institute of Medical Sciences, School of Medicine, Medical Sciences and Nutrition, University of Aberdeen, Aberdeen AB25 2ZD, UK

**Keywords:** Cancer biology, Drug discovery, Early-career researchers, Meeting review

## Abstract

The inaugural ‘British Association of Cancer Research (BACR) Early Career Conference, Trailblazers in Cancer Research 2023’, was a 2-day meeting held in Manchester, UK. Recognising the disruption caused by the COVID-19 pandemic to early-career researchers (ECRs), the BACR executive committee organised an in-person conference to address the lack of network and training opportunities during this time. The conference brought together PhD students and post-doctoral researchers from across the UK and beyond, who shared their outstanding contributions to cancer research. The meeting incorporated several cutting-edge cancer themes, including ‘Cancer Cell Signalling and The Tumour Microenvironment’; ‘Emerging Approaches in Cancer Treatment’; ‘Cancer Omics and Lifestyle’, and ‘Nutrition and Cancer’. Alongside showcasing world-class cancer research, the meeting included a career-focused session which allowed industrial and non-academic speakers to provide vital insight into alternative career paths aside from the familiar ‘academic’ route. Importantly, the conference also introduced delegates to Patient Public Involvement in cancer research, an area of limited experience for many. Overall, the BACR Trailblazers Conference was hugely successful and presented an excellent platform for collaboration and networking among ECRs in cancer research.

## Teaser

**Summary:** The British Association of Cancer Research Early Career Conference, Trailblazers in Cancer Research, brought together researchers from across the UK and beyond to share their outstanding contributions to cancer research.

## Introduction

The British Association of Cancer Research (BACR) is the largest British-based cancer society, with over 1400 members. Founded in 1960 by the vision of Sir Alexander Haddow, then Director of the Chester Beatty Research Institute, the BACR is now entering its 64th year. The mission statement is to “Promote the advance of research in relation to all aspects of cancer and encourage the exchange of information”. This is achieved through disseminating and exchanging information through conferences and meetings, bringing together world-class experts from various sectors related to cancer research to progress our understanding of cancer and its treatment. At the core of the BACR community are early-career researchers (ECRs). ECRs arguably suffered the most during the COVID-19 pandemic (Woolston, 2021). Recognising this, the BACR executive committee decided to organise an in-person conference specifically aimed at the needs of ECRs in the post-COVID-19 era. ECR BACR members were invited to express interest in being co-opted into the BACR executive to provide leadership in organising this inaugural meeting. Following interview, Dr Francis M. Barnieh was selected, who formed an ECR committee consisting of cancer researchers from across the UK and at varying career stages (Mr Kyle Greenland, Dr Kathryn Pennel, Dr Giorgia Cioccoloni and Dr Connor Rogerson), with Professor Valerie Speirs (BACR Honorary secretary) providing academic oversight. The ECR stage is a critical juncture in a researcher's development to generate academic networks that help build towards research independence (Ansmann et al., 2014; Levine and Rathmell, 2020). Opportunities for ECRs to present their work outside of their host Institutes remain rare and coveted. Moreover, evidence suggests that rates of new collaborations have declined following the COVID-19 pandemic (Gao et al., 2021). To address this, a 2-day residential meeting was organised under the umbrella of BACR to provide opportunities for ECRs to present their work, establish new networks and learn from world-leading cancer researchers. Inspiring the next generation is essential to advance cancer research and translate scientific findings to better prevent, diagnose, and treat cancer, resulting in improved outcomes for patients. To make this accessible to as many ECRs as possible, BACR absorbed most of the cost, with a nominal all-inclusive attendance fee (£100/delegate).

### The conference

**Fig. 1.**
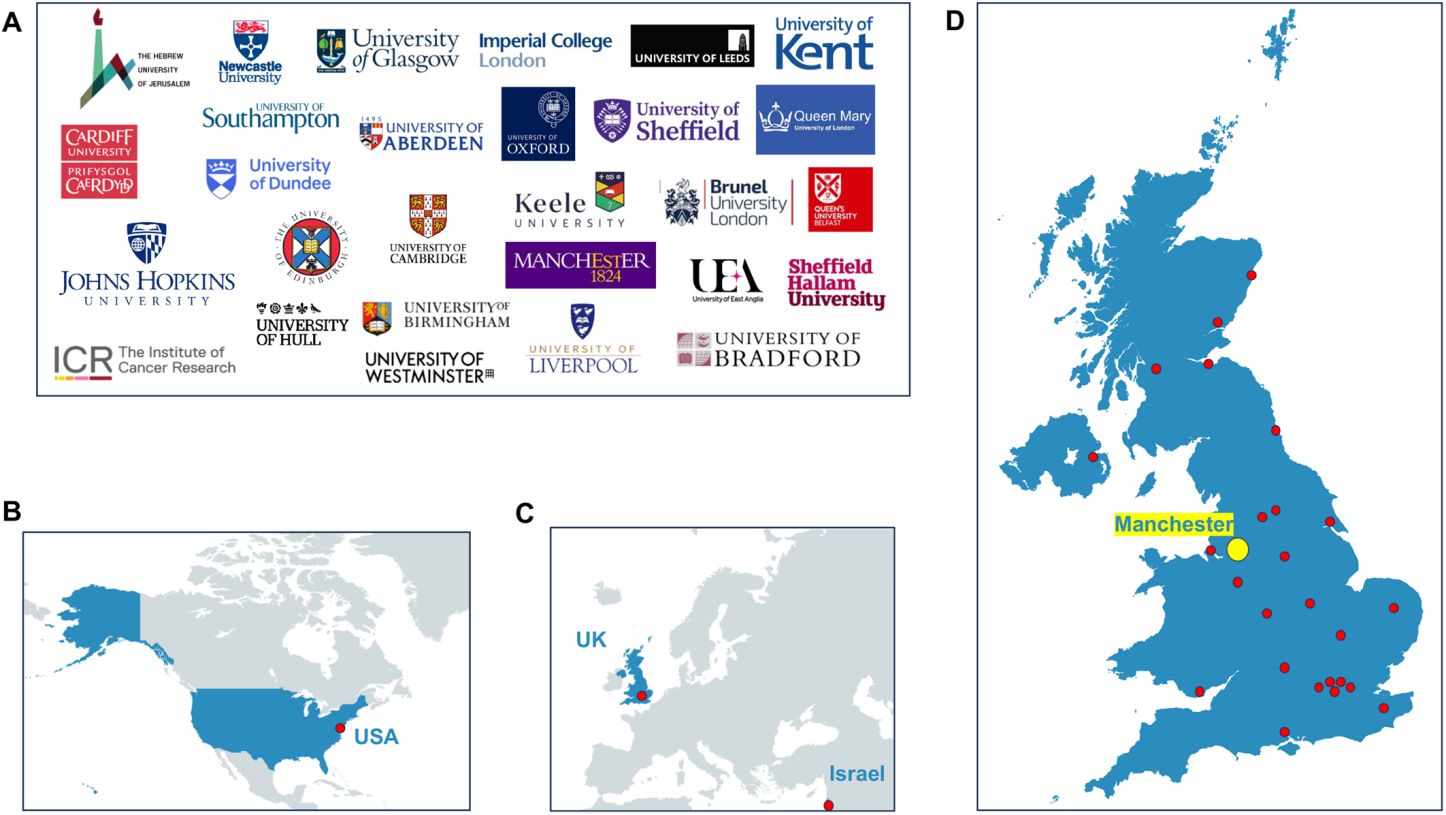
**Geographical distribution of academic institutions represented at The British Association of Cancer Research (BACR) Early Career Researcher (ECR) Conference on the 20th – 21st of June 2023 in Manchester, UK.** (A) Logos representing academic institutes of delegates that attended the ECR conference. Graphical distribution of delegates from (B) North America, (C) Europe and Asia and (D) across the UK. The red dots represent individual academic institutions. The yellow dot indicates the location of the conference, Manchester, UK.

**Fig. 2.**
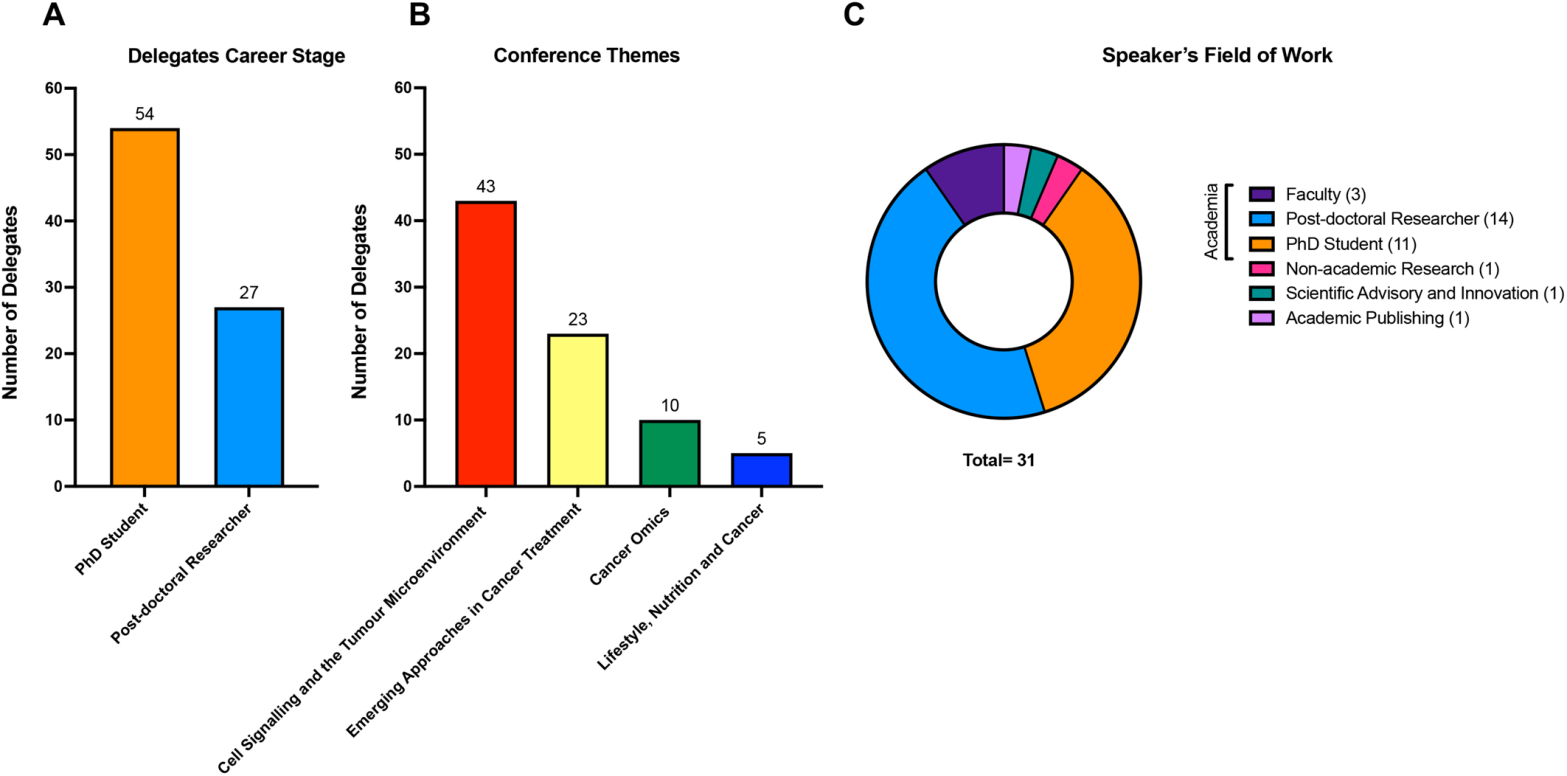
**Distribution of delegates' career stage, primary research theme and speaker profession.** Chart showcasing (A) the career stage of delegates attending, (B) the primary research themes of abstracts submitted by delegates and (C) the speaker's field of work.

Trailblazers took place from 20th –21st of June 2023, with 81 ECRs (54 PhD students; 27 post-doctoral researchers), gathering in Manchester, UK (Fig. 1A). Delegates shared their outstanding contributions in cancer research through 14 oral talks, 81 posters, plus informal networking. Although predominantly UK-based, delegates travelled from as far afield as Israel and the US, demonstrating the international reach that the BACR has (Fig. 1B-D). Conference delegates were treated to two inspirational keynote lectures delivered by Professor Michelle Garrett (University of Kent, UK) and Professor Chris Bakal (Institute of Cancer Research). Professor Garrett reflected on her diverse career history encompassing academia and industry and discussed her pivotal role in the discovery and preclinical investigation of CCT128930, a key prototype drug that was later modified to capivasertib (AZD5363), a potential first-in-class AKT inhibitor (Turner et al., 2023; Yap et al., 2011). She highlighted the importance of assembling diverse scientific teams during the drug discovery process, with expertise required in a broad range of scientific disciplines, from physics to molecular biology. Professor Bakal delivered a fascinating talk on the future of cancer research, including his pioneering contributions in artificial intelligence (AI), particularly in probing cancer cells' shape-shifting abilities (Barker et al., 2022). In addition, a cancer survivor and academic, Dr Natalie Yates-Bolton, introduced ECRs to her experience as a cancer patient and affirmed the importance of patient involvement in their research design. This was new to many of the delegates and emphasised the need for researchers to remember the reasons behind their work and the translational potential of their findings. A special moment was Natalie's realisation that Professor Garrett developed her chemotherapy treatment (Palbociclib) during her time at Onyx Pharmaceuticals. The scientific program was split into four cutting-edge cancer themes across 2 days, in which delegates presented their research work in the form of selected oral or poster presentations. Themes included cancer cell signalling and the tumour microenvironment; emerging approaches in cancer treatment; cancer omics and lifestyle, nutrition and cancer. All abstracts submitted by delegates were reviewed by the ECR committee, with a select few chosen to present their research based on the novelty, impact, and relevance to the conference themes (Fig. 2A-C).

**Fig. 3.**
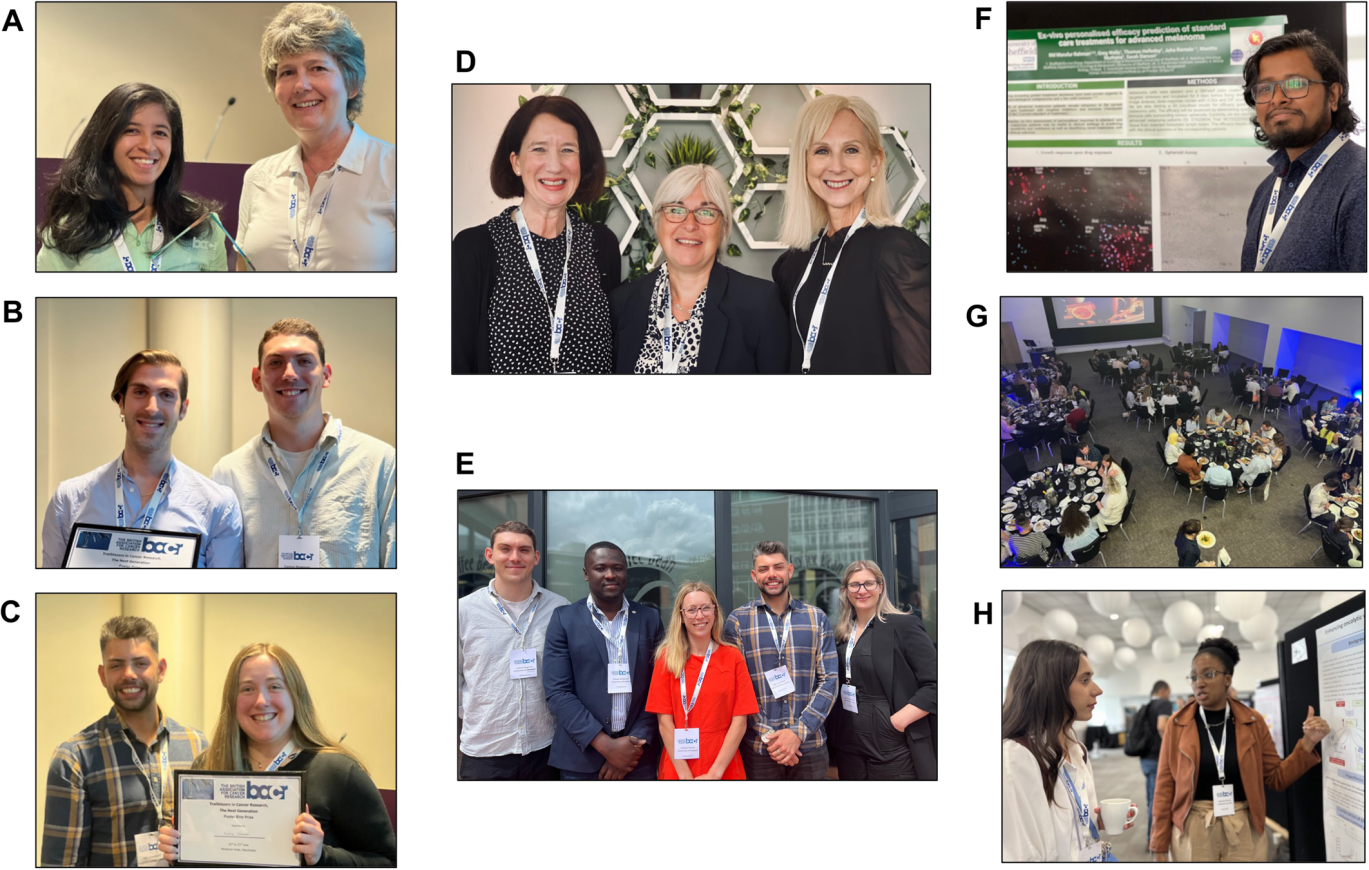
**Pictures from The British Association of Cancer Research (BACR) Early Career Researcher (ECR) Conference.** (A) Ms Vasudha Tandon was awarded the best oral presentation pictured alongside Professor Valerie Speirs. (B) Mr Fabrizio Fai received the poster award, pictured alongside Dr Connor Rogerson. (C) Dr Katie Hanna was awarded the prize for the best poster-blitz presentation, pictured alongside Mr Kyle Greenland. (D) Professor Michelle Garrett, Professor Ingunn Holen (BACR Chair) and Dr Natalie Yates-Bolton are pictured following the conference plenary talks. (E) The BACR ECR conference organising committee (L-R; Dr Connor Rogerson, Dr Francis M Barnieh, Dr Kathryn Pennel, Mr Kyle Greenland and Dr Giorgia Cioccoloni). (F) Dr Marufur Rahman presenting his findings during the poster session. (G) Conference dinner concluding the first day. (H) Two ECRs discussing their research findings during a poster presentation. Images courtesy of Barbora Gaborova.

### Cellular signalling and tumour microenvironment session

The first session focused on cellular signalling and the tumour microenvironment, chaired by Dr Kathryn Pennel (University of Glasgow, UK). This was the first time that she had chaired a session at a conference of this magnitude and was “…an incredible opportunity to introduce such a fantastic range of early career researchers.” and “…an exciting and rare chance to chair a session of this size.”. The session began with a talk from Dr Jiabou Zhou, a recent PhD graduate from the University of Sheffield, delivering a presentation on the role of interleukin 1 beta (IL-1β) in breast cancer bone metastases. Dr Zhou discussed the pleiotropic roles of IL-1β and presented research showing IL-1β inhibition both prevents and promotes metastases depending on the anatomical site. He finished by showing data that highlighted the pro- and anti-tumour effects of IL-1β on different inflammatory populations within primary breast cancer tumours. The second speaker was PhD student Esra Ermis Akyuz (University of Leeds, UK) who described the emerging roles of tumour suppressor gene *CUB* and Sushi Multiple Domains 1 (*CMSD1*) in cancer. Data from her PhD research thus far has explored *CMSD1* knockdown, which implicated *CMSD1* in breast cancer cell proliferation, invasion, and identified a novel association with proteins related to epithelial to mesenchymal transition. She posted on social media after the conference that the event was “…fantastic and the friendliest conference I have ever attended.”. Next, post-doctoral researcher Dr Emily Webb (University of Edinburgh, UK) delivered an excellent presentation on her work on Kindlin-1 in breast cancer including its prominent role in the anti-cancer immune response and discussed data exploring focal adhesion kinase and integrin-linked kinase in glioblastoma and their effects on T-lymphocyte and macrophage infiltration within the tumour microenvironment. Dr Webb gave a succinct overview of immune cell lineages, which was expertly explained for the non-immunologist delegates. Dr Anna Richards (Cardiff University, UK) rounded off the session with a talk exploring TNF-related apoptosis-inducing ligand signalling in pancreatic ductal adenocarcinoma (PDAC). She discussed the therapeutic potential of inhibiting intermediate pathway component FLIP as a mechanism to reactivate apoptosis in PDAC with data from a range of *in vitro* and *in vivo* models. After the conference, Dr Richards posted on social media that she “…had a great experience presenting.”. This session generated a high degree of interest from the audience, with every presenter asked multiple questions by peers. The topics covered were discussed informally during networking breaks between delegates with similar interests, and potential new collaborations were developed.

### Cancer omics

The second session was ‘Cancer Omics’ to provide an outlet for the many emerging ‘omics’ technologies that are now being used to understand cancer at the molecular level. Dr Connor Rogerson (University of Cambridge, UK) was honoured to chair this and was impressed by the high-quality research from three excellent oral presentations, showcasing a wealth of omics technologies and their applications in interrogating various cancer types. Dr Rayzel Fernandes (post-doctoral researcher, Imperial College London, UK) delivered an intriguing talk about the role of novel enhancer RNAs and their role in prostate cancer. Using GRO-seq, she identified numerous enhancer RNAs that were differentially expressed in therapeutic-resistant cell lines compared to their parental treatment-sensitive pairs. Moreover, by knocking down these enhancer RNAs using small interfering RNA (siRNAs), she was able to slow cellular proliferation. The next speaker was Dr Conrado Quiles, [post-doctoral researcher, Cancer Research UK (CRUK) Manchester Centre]. He used proteomics and RNA sequencing to gain insight into the role of hypoxia extra-cellular matrix (ECM) remodelling in bladder cancer. He showed differences between the mRNA transcript and proteomics of ECM generated under different oxygen concentrations. The final speaker was Narmeen Daher, a PhD student at the Hebrew University of Jerusalem, Israel. She spoke on her research on oral squamous cell carcinoma optimising therapies for patients with radio-resistant tumours using naïve patient-derived xenografts and profiling cell surface markers. Post-radiotherapy, drastic changes in cell surface markers of patients who responded to radiotherapy were seen and are being used to stratify patient treatment regimens. The Cancer Omics session sparked curiosity about the rapid use of omics in cancer research and fostered collaborations between researchers keen to implement these technologies in their own research.

### Emerging approaches in cancer treatment

The first session of day two was chaired by Dr Francis M Barnieh and centred on emerging insights into the development of novel cancer therapeutics. Dr Daniel Turnham, (research associate, Cardiff University, UK), opened with a talk on a novel antibody-drug conjugate which exploits the high expression of receptor-for-advanced-glycation-end-products (RAGE) in metastatic prostate cancer. His work contributes to the growing interest in the use of antibody-based therapeutics as tools for precision oncology. The next speaker was Dr Abiola Ayanlaja, a post-doctoral research fellow (John Hopkins University, USA), who delivered a presentation on targeting SHP2-dependent adaptive resistance to BRAF and MEK inhibition in glioma. Using MEK, BRAF and SHP2 inhibitors, he demonstrated the potential therapeutic advantage of combined BRAF and SHP2 inhibition in MEKi-resistant BRAF mutant glioma preclinical models. This work presented new insights into addressing the high incidence of MEKi resistance in paediatric gliomas. Dr Ameera Jailani, (post-doctoral research associate, University of Sheffield, UK), followed with a talk on her work “…targeting the adrenomedullin-2 receptor in prostate cancer”. Using preclinical prostate cancer models, Ameera demonstrated the therapeutic potential of adrenomedullin-2 targeting in and described the development of a novel small molecule antagonist against the adrenomedullin-2 receptor with a significant antitumor effect. To conclude the session, final year PhD student Ms Vasudha Tando (University of Dundee, UK), presented her work on the development of a brain penetrant PI3 K/WNT pathway inhibitor for the treatment of glioblastoma. In preclinical models of glioblastoma, the molecule DYR726, was shown to be a specific and selective kinase inhibitor with a significant therapeutic window, which surpassed those exhibited by multiple other FDA-approved kinase inhibitors. This captivating talk was awarded Best Oral Presentation of the conference, selected by the BACR Executive. Overall, this session was insightful and thought-provoking that demonstrated the commitment of ECRs in developing novel therapeutics for cancer treatment.

### Lifestyle, nutrition, and cancer

The last session focused on Lifestyle, Nutrition, and Cancer, and was chaired by Dr Giorgia Cioccoloni (University of Leeds, UK). She emphasized that “Trailblazers was a big opportunity for the ECR community to showcase their research and talent while fostering connections with other future leaders in cancer research.”. The session featured three brilliant speakers and provided an exceptional platform to highlight new discoveries on the intricate relationship between diet and cancer, which is often negated. The first speaker was Dr Fiona C. Malcomson, recently appointed as Lecturer in Human Nutrition (Newcastle University, UK). She presented her work on the associations between adherence to the World Cancer Research Fund (WCRF)/American Institute for Cancer Research (AICR) cancer prevention recommendations and the risk of several types of cancer in a UK cohort of 94,778 participants. Her findings shed light on the significant impact of dietary choices on cancer risk. Ms Non Williams, (PhD student, Cardiff University, UK), then delivered an outstanding presentation on the role of diet and lifestyle in the regulation of intestinal stem cells and their impact on cancer risk. Her research revealed that a high-fat diet can increase intestinal stem cell (ISC) number and size, while exercise can counteract this expansion. Furthermore, she uncovered that a novel anti-obesity drug may have unexpected pro-tumorigenic effects, as it increased ISC number and size. Ms Williams took to social media to describe her experience, “I had a great time presenting my data at the BACR Trailblazers conference. Thank you to the organizers for arranging such an excellent event!”. The final speaker in this session was Dr Hannah Harrison, (post-doctoral researcher, University of Manchester, UK). Dr Harrison discussed a new *in vitro* model of breast cancer metastasis and niche priming, a model that could replace animal testing for studying cancer progression, holding promise for observing interactions between different cancer sites, potentially changing cancer research methodology. Overall, this session provided the audience with a fresh and unique perspective on cancer research. The diverse range of topics presented by the speakers and poster presenters demonstrated the exciting advancements in scientific knowledge within the field. The session was a success, setting the stage for new collaborations and the prospect of innovative and ground-breaking research in the years to come.

### Poster presentations and networking opportunities

As the COVID-19 pandemic limited opportunities for ECRs to present their research, four poster sessions, covering 81 posters, were included as well as the talks, providing ECRs with limited experience an ideal introduction to presenting at scientific conferences as well as ample time for networking in a welcoming and less intimidating environment than typical scientific conferences. Eight posters were selected for an entertaining Poster Blitz session, chaired by Mr Kyle Greenland, where presenters had 5 min to describe their research, with post-doctoral scientist, Dr Katie Hanna (University of Aberdeen, UK), chosen as the winner by fellow ECRs using online voting. Mr Fabrizio Fai (Queen Mary University of London, UK) received the best poster award, judged by the ECR organising committee. Delegates also had the opportunity to engage with representatives from companies that provided sponsorship for the event, during refreshment breaks to see first-hand some of the technologies they might employ in their research.

### Expanding horizons on careers outside of academia

Not all scientists follow an academic career path, and ECRs working in academic institutions often don't appreciate what other careers may offer post PhD. The inclusion of a career-focused session in the programme allowed industrial and non-academic talks from Dr Rachel Eyre (NC3Rs Programme Manager, UK), Dr Claire Baron (Charles River Laboratories, UK) and Dr Diana Romero (Nature Publishing Group). These provided valuable insights into alternative career paths aside from the familiar ‘academic’ route, highlighting the broad range of career options available to scientists outside of academia, which many of us had perhaps not considered before.

### Reflections on the conference

To wrap up a successful and inspirational meeting, Professor Valerie Speirs closed the conference commenting on the high-quality research and enthusiastic buzz of conversation throughout the event. Dr Hanna later reflected on the experience, saying, “I was humbled to have been selected as the winner of the poster blitz prize. Presenting at this conference, receiving this award, and having inspirational conversations with other ECRs gave me the motivation and confidence to defend my PhD at my recent viva successfully.”. Ms Tandon echoed the value and importance of networking at the conference by saying, “I am incredibly honoured to have received the prize for best Oral Presentation for presenting my PhD work at BACR's Trailblazers in Cancer Research conference. Moreover, I am extremely thankful to the committee, as many amazing, thought-provoking talks were given throughout the conference. Winning this award led to many more meaningful networking connections and job opportunities, and I am excited to put it on my CV!”.

### Conclusions

Overall, the BACR Trailblazers Conference was well received by delegates. It provided an excellent platform for collaborative discussion and networking among ECRs in cancer research, as well as providing the organisers with valuable insight into what goes into conference organisation. This has now created a platform (with support from BACR) to build a wider network of ECRs working in cancer research across the UK and beyond. This will help provide a professional support network outside of our own Institutes through virtual events, such as webinars on careers in science and grant/fellowship writing. The ECR committee look forward to seeing how the new connections made at the conference are maintained and translate into cross-institution collaboration.

## Footnote

For more information about the activities of the British Association of Cancer Research, including upcoming meetings, awards, and travel bursaries, please visit the website: https://www.bacr.org.uk.
